# Genetic landscape of atopic dermatitis

**DOI:** 10.1097/ACI.0000000000001005

**Published:** 2024-06-24

**Authors:** Lavinia Paternoster

**Affiliations:** MRC Integrative Epidemiology Unit, Bristol Medical School, University of Bristol, Bristol, UK; NIHR Bristol Biomedical Research Centre, University Hospital Bristol and Weston NHS Foundation Trust and University of Bristol, Bristol, UK

**Keywords:** Atopic dermatitis, genetics, rare variants, GWAS, Mendelian Randomization

## Abstract

**Purpose of Review:**

This review summarises recent advances in identifying genetic risk factors for atopic dermatitis (AD) and how these genetic associations are being used to explore the causal relationships between atopic dermatitis and potential risk factors and downstream outcomes.

**Recent Findings:**

A recent large-scale GWAS meta-analysis has identified 91 genetic loci associated with AD. Rare variant studies have also identified new gain- or loss-of-function variants implicated in AD, particularly for *FLG* and *STAT6/JAK1*. Finally, there has been a surge in utilising genetic association data to investigate the causal relationships between AD and other traits. Mendelian Randomization studies have found that various metabolites and gut microbiota are causal for AD and have causally implicate AD in the development of alopecia areata, diabetes, vascular dementia and some cancers.

## Introduction

It has long been known that atopic dermatitis (AD) is highly heritable [1]. Since FLG was the first gene implicated for AD, the past 15 years has seen advances in genotyping technology and Biobank data collection, which has resulted in studies identifying many genomic loci associated with AD.

### New genetic variants identified in GWAS

The largest GWAS meta-analysis for AD was published last year [2**]. In that study we analysed genetic data from >4 million participants to identify 91 genetic loci associated with AD, 32 of these being novel. Downstream analyses of this set of GWAS signals showed enrichment for pathways involved in immune system regulation and activation.

As more genetic associations for AD emerge, it is also becoming clearer how AD is genetically related to other traits. For example, a recent study showed that genes shared between AD and autoimmune disease cluster in Th1, Th2, Th17 pathways [3*]. In our GWAS study we also identified notable genetic correlations with depression/anxiety and gastritis (in addition to the expected allergic traits) [2**].

### Rare variants

Whilst GWAS focus on common variants, many studies aim to identify rare variants. *FLG* has been established as an AD risk factor for a long time, but new variants are still being discovered. A recent step-change in genotyping *FLG* was achieved using Oxford Nanopore long-read sequencing (as opposed to short-read sequencing), resulting in increased ability to identify copy number variation, more accurate phasing and repeat-specific locations for *FLG* loss of function variants [4*].

*STAT6*, previously implicated in AD GWAS [5, 6] has recently gained attention as a source of rare gain-of-function (GOF) variants that result in a severe, early-onset broad allergic disease phenotype. Across five studies, 21 individuals harbouring 11 different *STAT6* GOF variants have been identified [7**, 8**, 9**, 10**, 11**]. STAT6 is part of the IL4R/JAK/STAT6 pathway and consistent with this, *STAT6* GOF patients have responded well to anti-IL4 antibody dupilumab and JAK-inhibitors. Given this, it has been suggested that patients with severe early-onset allergic disease may benefit from early STAT6 GOF assessment [12].

Rare *JAK1* GOF variants have also been identified in families with a broad and severe inflammatory phenotype, including C787F identified in 9 affected individuals of one family [13**]. Another recent study identified 59 individuals with 4 novel *JAK1* GOF variants, showing a more common autoimmune and inflammatory disease manifestation than has been reported previously [14**]. Consistent with *STAT6*, there is preliminary evidence that treatment of these patients with JAK-inhibitors may be especially effective, and warrant early identification and a precision medicine approach to treatment.

A dominant rare variant has also recently been identified in *CARD11* in a young patient (of Italian descent) with severe atopic dermatitis, food induced anaphylaxis and hypogammaglobulinemia [15*]. Common variants predicted to affect this gene have also been previously implicated in AD GWAS, but interestingly, only in Japanese individuals [5, 16].

### Differences in genetic effects by ancestry

Individuals of non-European descent are currently disproportionately under-represented in genetic studies of AD. Therefore, differences in the genetic aetiology of AD between individuals of different ancestry, could result in large inequalities in terms of available effective treatments that target the underlying causes of disease. This has been seen for cardiovascular disease, where several drugs are less (or in-)effective in people of colour [17].

There is some reassuring evidence from our recent GWAS, that although the index SNPs association may not always replicate between ancestral groups, gene-based or region-based comparisons suggest far fewer differences exist, with only 2 of the 91 associations showing strong evidence of population-specific association (rs4312054 and rs9864845, which appear to be specific to East Asians) [2**].

It has long been reported that there may ethnic differences in the role of *FLG* in AD, with mutations observed in Europeans being found at much lower frequencies (or entirely absent) in individuals of Asian and African descent [18, 19]. New evidence is starting to fill the gap of knowledge of *FLG* variants in those of African-descent. Studies in Brazil (with high African ancestry) have investigated the frequency and AD association of *FLG* variants [20*, 21*] and a US-based study report 20-shared and 10 African ancestry specific (1 novel) *FLG* variants [22*].

When differences are seen at the variant level, but not at the gene level, this can be because the populations share a causal variant, but this getting tagged differently in the two populations (due to differences in linkage disequilibrium), or the causal variants exist at different frequencies, or different causal mutations have arisen in ancestrally separate groups. The first situation is less of a concern, but the latter two, could result in differences of magnitude in efficacy of drugs that target these genes. Therefore, further careful analysis comparing genetic associations in different population groups is necessary to ensure effective and equitable drug design.

### Post-GWAS functional studies

Identification of a SNP, or region associated with a trait in a GWAS requires follow-up to identify the implicated gene (as well as functional mechanism and the relevant cell type). In our recent GWAS, we follow the GWAS with a bioinformatic pipeline to integrate evidence from a wide variety of sources and prioritise genes as the likely causal candidates at each locus.

Several other recent studies have attempted to identify the likely candidate gene from GWAS and validate/functionally characterise these through experimental work. Investigation of one GWAS hit (at 9q21.11) intronic to *TJP2*, found strong evidence that the GWAS SNP was associated with *TJP2* expression, and further investigation suggested that methylation of a nearby CpG site could provide the functional mechanism [23*]. The same group also similarly characterised a genetic association at *LOC100294145* with bioinformatic and in vitro analyses [24*]. A new bioinformatic method, maxATAC, which aims to predict transcription factor binding sites using ATAC-Seq data, demonstrated their method in AD [25*]. They found that transcription factors MYB and FOXP1 are predicted to be associated with allele dependent chromatin accessibility at AD loci, adding another dimension to how GWAS loci can be functionally followed up with in silico analyses. Another statistical approach to identify causal genes recently employed in AD is to combine the genetic data with transcriptomic data to directly determine the genes whose expression has a causal effect on AD [26]. This identified four novel genes for AD: *AQP3, PDCD1, ADCY3, and DOLPP1*.

As a demonstration of how the function of a gene can then be further characterised experimentally, a recent paper has used CRISPR/Cas9 technology to generate *FLG* knock-out keratinocytes for causal functional characterisation of this gene [27**]. This technology is likely to become more widely used for functional characterisation of implicated genes, though identifying the right model can be a barrier for such investigations.

### Genetically informed drug targets

It has been shown that genetic evidence (such as from GWAS) implicating a target gene in the pathogenesis of a disease leads to higher success rate in clinical trials [28*]. The recently approved, Lebrikizumab [29], is supported by genetic associations with *IL4, KIF3A* and *IL13*. However, dermatology may have not yet reached the same potential as other disease areas in utilising genetic data for efficient drug discovery [28*] and so it will be interesting to see if over the next few years more genetically supported AD drugs emerge.

### Predictive ability of genetic risk factors

The vast majority of genetic associations for AD have very small effect sizes, but can be combined together into a polygenic risk score that captures more genetic risk. Previously a study looking at the predictive ability of genetics for AD, found that using a broad allergy PRS incorporating *FLG*, the predictive ability is promising (area under the curve (AUC)=0.76, rising to 0.80 with the inclusion of age and sex) [30]. In a recent extension of this, an atopic PRS was found to be associated with paradoxical eczema developing in psoriasis patients treated with biologics, but the predictive ability of this has not yet reached clinical significance (maximum AUC=0.69) [31*].

### Digging deeper into the AD phenotype

Following the success in identifying genes implicated in AD susceptibility, attention is now turning to related phenotypes to get a deeper understanding of AD aetiology. Longitudinal clustering methods have been utilised to allow for genetic association testing with longitudinal patterns of allergic disease [32*]. GWAS of related trait IgE has also recently been performed, identifying eight genome-wide significant variants [33*] and whilst *FLG* genotype has significant impact on AD susceptibility, it has been shown that it does not affect response to dupilumab treatment [34*]. Further refinement of phenotypes will help build a complete picture of how genetic factors influence AD and subsequent progression of the disease.

### Utilising genetic data to explore causal relationships

**Figure 1:**
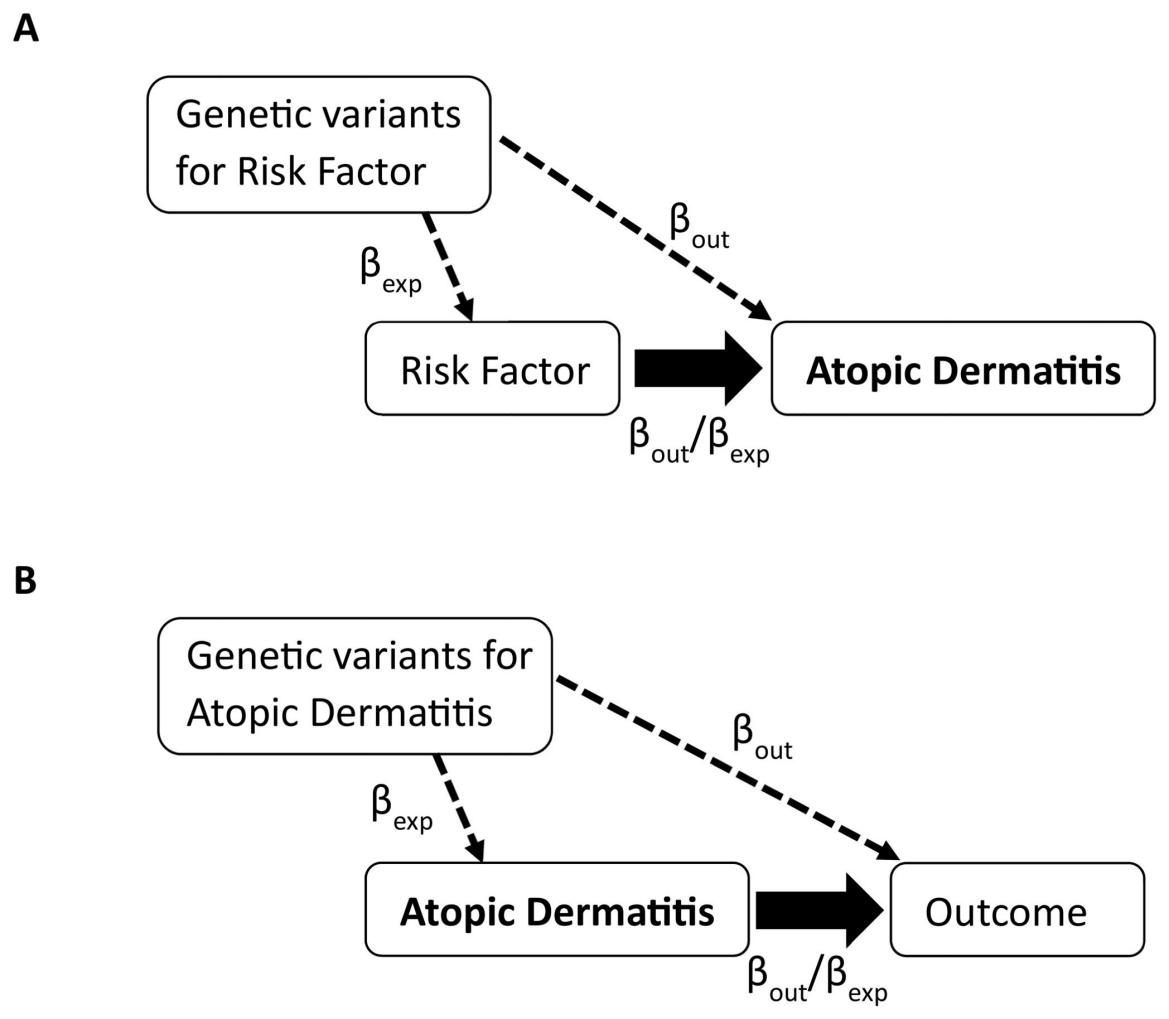
Schematic of Mendelian Randomization analysis involving AD (A) Testing for the causal effect of potential risk factors on AD, and (B) Testing for the causal effect of AD on downstream outcomes. In both cases the bold arrow is the causal effect that is being estimated. Genetic risk factors (instruments) for the relevant exposure are selected (and the association recorded, β_exp,_) and then the association of these instruments is obtained for the outcome (β_out_). Then the wald ratio [β_out_/β_exp_] can be calculated to estimate the causal effect of the exposure on the outcome.

With the increasing availability of large-scale openly accessible AD GWAS data, there has been a surge in utilising this data to investigate causality for many AD epidemiological relationships with an approach called Mendelian Randomization (MR). MR uses genetic proxies for exposures of interest and tests for an association between these genetic proxies and outcomes (figure 1). In this context, GWAS data for AD is combined with GWAS of other traits to determine if AD is causal for certain outcomes, or if particular risk factors have a causal effect on AD. A recent study conducted a systematic review of 30 MR studies in AD [35**], finding evidence for body mass index, gut microbial flora, the IL-18 signalling pathway and gastroesophageal reflux disease as causal risk factors for AD. They also reported that genetic evidence shows that AD is causal for several disease outcomes including heart failure, rheumatoid arthritis and conjunctivitis [35**]. Indicative of the popularity of this statistical technique, in the past 18 months >20 additional AD MR studies have been published. This has included exploring the causal role of metabolites in disease, with one study reporting evidence that increased TNFS14 (and decreased DHA) in the blood are causal risk factors for AD [36*] and another showing that reduced urinary tyrosine [37*] is causal for AD, as well as further studies reporting the causal role of specific gut microbiota [38*, 39*, 40*]. An even greater number of studies have claimed evidence that genetic liability to AD is causal for later health outcomes (Figure 1B), including Type 1 and Type 2 diabetes [41*], basal cell carcinoma and cutaneous squamous cell carcinoma [42*], alopecia areata [43*], vascular dementia [44*], allergic rhinitis [45*] and increased caudate volume [46*], as well as reduced Parkinson’s disease risk [47*]. MR has also been used to refute causal relationships for some observational associations, such as between AD and COVID-19 [48*, 49*]. There are important limitations of this approach to be considered when undertaking or interpreting such studies and STROBE-MR reporting guidelines have been developed to mitigate these [50]. One assumption of MR is that variants do not have a pleiotropic effect on the outcome (i.e. the genetic variants used to proxy the exposure do not affect the outcome through a path independent of the exposure). This is difficult assumption to test, but several methods exist that attempt to estimate and account for pleiotropy. Violation of this assumption can also manifest in situations where an underlying biological impact of the variant affects two diseases independently. This is a particular concern when the causal effect appears to go in both directions, as has been observed for AD and ADHD [51], inflammatory bowel disease [52], asthma [53] and chronic kidney disease [54]. Another way of thinking of this is that the genetic instrument used is not specific for the exposure trait. This should be investigated in studies and Steiger filtering is one approach to avoid this pitfall [55]. Null studies must also be reported carefully, as some MR estimates can have wide confidence intervals, which while do not provide any evidence for a causal effect, often also do not rule out quite large causal effects, as was reported for glaucoma, where confidence intervals for a causal odds ratio of AD on glaucoma ranged from 0.95 to 1.27 [56] and ADHD 0.93 to 1.11 [57]. As larger GWAS become available, statistical power for these research questions will increase. Given the paucity of large GWAS in individuals of non-European ancestry, the current MR studies also suffer from lack of generalisability. However, recently an MR study was able to compare the causal effect of AD on esophageal cancer in Europeans and East Asians, finding causal evidence only in the latter [58].

## Conclusion

In this paper I have outlined the key recent advances in the genetic aetiology of AD. Whilst the list of genes implicated in the disease has grown substantially, the field now moves into a more challenging phase, where nuance of how genetic associations functionally affect the underlying molecular pathways, and how consistent genetic associations are between individuals of different ancestry is necessary.

## Summary

The past year has seen a huge increase in the genes implicated for AD and in the use of genetics to explore causal relationships. The latter requires caution in implementation and interpretation, but is a promising area of research. In the coming years, increasing the ethnic diversity of AD genetic studies would be very welcome and the translation of current genetic findings into new drugs will be an exciting area of development.

## Key points

Recent large-scale GWAS have identified a large number of genetic variants associated with atopic dermatitis

Functional follow-up studies to characterise these genetic associations, particularly to identify the causal gene, relevant tissue and mechanism of perturbation, are emerging and will be critical to ensure translation of findings.

Genetic studies of diverse cohorts have generally demonstrated that at the gene level there is huge consistency in genetic effects between populations, but at the variant level there can be significant variability.

GWAS resources are now being utilised to undertake Mendelian Randomization studies to investigate causal relationships between AD and potential causes and consequences.
